# Isocoumarins: a new class of selective carbonic anhydrase IX and XII inhibitors

**DOI:** 10.1080/14756366.2022.2041630

**Published:** 2022-02-21

**Authors:** Mehmet Onyılmaz, Murat Koca, Alessandro Bonardi, Mustafa Degirmenci, Claudiu T. Supuran

**Affiliations:** a Faculty of Science and Arts, Department of Chemistry, Harran University, Şanlıurfa, Turkey; b Department of Pharmaceutical Chemistry, Faculty of Pharmacy, Adıyaman University, Adıyaman, Turkey; c Department of Neurofarba, Sezione di Scienze Farmaceutiche, Università degli Studi di Firenze, Sesto Fiorentino, Italy

**Keywords:** Carbonic anhydrase, inhibitors, isocoumarin, carboxy-phenylacetic aldehydes

## Abstract

Isocoumarins, isomeric to comarins which act as effective carbonic anhydrase (CA, EC 4.2.1.1) inhibitors, were investigated for the first time as inhibitors of this enzyme. A series of 3-substituted and 3,4-disubstituted isocoumarins incorporating phenylhydrazone, 1-phenyl-pyrazole and pyrazolo-substituted pyrimidine trione/thioxo-pyrimidine dione moieties were investigated for their interaction with four human (h) CA isoforms, hCA I, II, IX and XII, known to be important drug targets. hCA I and II were not inhibited by these compounds, whereas hCA IX and XII were inhibited in the low micromolar range by the less bulky derivatives. The inhibition constants ranged between 2.7–78.9 µM against hCA IX and of 1.2–66.5 µM against hCA XII. As for the coumarins, we hypothesise that the isocoumarins are hydrolysed by the esterase activity of the enzyme with formation of 2-carboxy-phenylacetic aldehydes which act as CA inhibitors. Isocoumarins represent a new class of CA inhibitors.

## 1. Introduction

**Figure 1.**
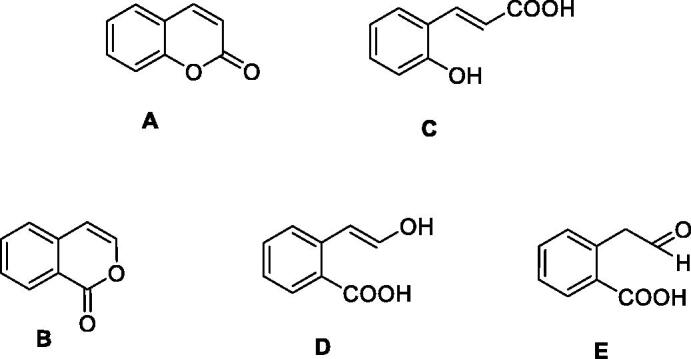
Coumarin (A) and isocoumarin (B), and their hydrolysis prroducts (C–E).

Isocoumarins, both naturally occurring^1^ and synthetic such derivatives^2^, similar to the isomeric comarins^3^, possess a multitude of applications in the drug design of pharmacologically relevant derivatives^3^^,^^4^. These two privileged scaffolds **A** and **B** (Figure 1) probably find many such applications due to the fact that the bicyclic ring system found in them combines a rather stable, planar aromatic scaffold with a good reactivity due to the lactone ring present in both derivatives, combined with the relative facility of derivatization at diverse pharmacophoric points with the possibility to generate new chemical space^1–4^. A salient feature of coumarins and isocoumarins is the relatively facile hydrolysis of their lactone ring with formation of 2-hydroxycinammic acid **C** (from coumarin) and 2-carboxy-phenylacetic aldehyde **E** from isocoumarins, as the enol **D** is unstable and spontaneously converts to **E**^1–4^ (Figure 1).

Coumarins were by far the most investigated class of such compounds, also because some of them are clinically used as anticoagulants for decades^5^ and were more recently investigated in detail as carbonic anhydrase (CA, EC 4.2.1.1) inhibitors^6^.

## 2. Materials and methods

### 2.1. General

All chemicals and anhydrous solvents were purchased from Sigma-Aldrich, Merck, Across Organics and TCI and used without further purification. Melting points (mp) were determined with SMP30 melting point apparatus in open capillaries and are uncorrected. FT-IR spectra were recorded by using Perkin Elmer Spectrum 100 FT-IR spectrometer. Nuclear Magnetic Resonance (^1^H-NMR and ^13^C-NMR) spectra of compounds were recorded using a an Agilent-NMR-vnmrs400 MHz and Bruker 300 MHz spectrometer in DMSO-d_6_ and TMS as an internal standard operating at 300 MHz for ^1^H-NMR and 75 MHz for ^13^C-NMR. Thin layer chromatography (TLC) was carried out on Merck silica gel 60 F_254_ plates.

### 2.2. General procedure for the synthesis of 3–(1-(2-phenylhydrazine) ethyl)-isochrom-1-one derivatives X(1–5)

The methyl ketone (10 mmol) and phenylhydrazine derivative compounds were added to a reaction flask by adding 20 ml EtOH with a catalytic amount of acetic acid and refluxed for 2 h. After the reaction complete, the obtained compounds were filtered off and crystallised from ethanol. The final products **X(1–5)** were dried under vacuum and fully characterised by FT-IR, ^1^H-NMR, ^13^C-NMR, and melting points.

**3–(1-(2-phenylhydrazono)ethyl)-1H-isochromen-1-one (X1)** Yield: 90%; mp: 202–204 °C; FT-IR (cm^−1^): 3294 (NH), 1695 (C = O); ^1^H-NMR (DMSO, δ, ppm): 2.21 (s, 3H), 6.94–7.69 (m, 10H), 8.28 (s, 1H).

**3–(1-(2–(4-chlorophenyl) hydrazono)ethyl)-1H-isochromen-1-one (X2)** Yield: 80%; mp: 238°–240 °C; FT-IR (cm^−1^): 3287 (NH), 1692 (C = O); ^1^H-NMR (DMSO, δ, ppm): 2.47 (s, 3H), 7.20–8.11 (m, 9H), 9.79 (s, 1H).

**4–(2-(1–(1-oxo-1H-isochromen-3-yl)ethylidene)hydrazinyl) benzonitrile (X3)** Yield: 82%; mp: 250°–252 °C; FT-IR (cm^−1^): 3271 (NH), 2222 (C≡N), 1692 (C = O), 1597 (C = N);^1^H-NMR (DMSO, δ, ppm): 2.45 (s, 3H), 7.29–8.12 (m, 9H), 10.18(s, 1H). ^13^C-NMR (DMSO, δ, ppm): 12.2, 82.9, 99.3, 101.1, 103.8, 113.8, 120.3, 120.4, 127.2, 129.2, 133.9, 135.7, 137.5, 137.7, 148.9, 152, 161.5, 175.4.

**4-methyl-3–(1-(2-phenylhydrazono)ethyl)-1H-isochromen-1-one (X4)** Yield: 82%; mp: 210°–212 °C; FT-IR (cm^−1^): 3271 (NH), 1733, 1709 (C = O); ^1^H-NMR (DMSO, δ, ppm): 2.17 (s, 3H), 2.47 (s, 3H), 7.18–8.17 (m, 9H), 9.57 (s, 1H). ^13^C-NMR (DMSO, δ, ppm): 13.4, 39.4, 109.3, 112.8, 113.4, 120.2, 124.6, 128.5, 129.3, 129.4, 135.6, 135.9, 138.9, 145.6, 149.6, 161.4.

**3–(1-(2–(4-chlorophenyl) hydrazono)ethyl)-4-methyl-1H-isochromen-1-one (X5)** Yield: 83%; mp: 240°–242 °C; FT-IR (cm^−1^): 3320 (NH), 1715 (C = O); ^1^H-NMR (DMSO, δ, ppm): 2.17 (s, 3H), 2.41 (s, 3H), 7.17–8.18 (m, 8H), 9.70 (s, 1H). ^13^C-NMR (DMSO, δ, ppm): 13.4, 40.6, 109.5, 114.8, 120.2, 123.5, 124.6, 128.6, 129.2, 129.3, 135.7, 136.8, 138.8, 144.6, 149.4, 161.3

### 2.3. General procedure for the synthesis of 3–(1-oxo-1H-isochromen-3-yl)-1-phenyl-pyrazole-4-carbaldehyde X(6–10) derivatives

The phenylhydrazone derivatives X (1–5) (10 mmol) and DMF (0.88 g, 12 mmol) were placed in a reaction flask and the POCl_3_ (1.84 g, 12 mmol) was added dropwise over the reaction mixture by keeping the temperature between 0° and 5 °C. After the completion of adding, the mixture was allowed to stir overnight at room temperature. Next day, the mixture was poured into the ice-water and it was triturated with 10% NaOH solution. The precipitate was filtered off and dried under vacuum at room temperature. The final products **X(6–10)** were fully characterised by FT-IR, ^1^H-NMR, ^13^C-NMR, and melting points.

**3–(1-oxo-1H-isochromen-3-yl)-1-phenyl-1H-pyrazole-4-carbaldehyde (X6)** Yield: 82%; mp: 228°–230 °C; FT-IR (cm^−1^): 3124, 2921 (C-H), 1728 (C = O isocoumarin), 1676 (C = O aldehyde); ^1^H-NMR (DMSO, δ, ppm): 7.22–8.32 (m, 10H), 8.53 (s, 1H), 10.54 (s, 1H).

**1–(4-chlorophenyl)-3–(1-oxo-1H-isochromen-3-yl)-1H-pyrazole-4-carbaldehyde (X7)** Yield: 80%; mp: 258°–260 °C; FT-IR (cm^−1^): 3287, 3025 (C-H), 1716 (C = O isocoumarin), 1682 (C = O aldehyde); ^1^H-NMR (DMSO, δ, ppm): 7.58–8.55 (m, 9H), 9.03 (s, 1H), 10.58 (s, 1H).

**4–(4-formyl-3–(1-oxo-1H-isochromen-3-yl)-1H-pyrazol-1-yl) benzonitrile (X8)** Yield: 78%; mp: >300^o^C; FT-IR (cm^−1^): 3120–3078 (CH), 2228 (C≡N), 1712 (C = O isocoumarin), 1673 (C = O aldehyde); ^1^H-NMR (DMSO, δ, ppm): 7.69–8.24 (m, 9H), 9.50 (s, 1H), 10.31 (s, 1H).

**3–(4-methyl-1-oxo-1H-isochromen-3-yl)-1-phenyl-1H-pyrazole-4-carbaldehyde (X9)** Yield: 80%; mp: 201°–203 °C; FT-IR (cm^−1^): 3117, 2921 (C-H), 1718 (C = O isocoumarin), 1679 (C = O aldehyde); ^1^H-NMR (DMSO, δ, ppm): 2.60 (s, 3H), 7.28–8.43 (m, 8H), 8.59 (s, 1H), 10.31 (s, 1H). ^13^C-NMR (DMSO, δ, ppm): 12.9, 113.5, 119.7, 121.1, 123.9, 124.7, 128.3, 128.8, 129.6, 129.8, 129.9, 134.9, 138.2, 138.8, 142.9, 146.8, 161.4, 185.5.

**1–(4-chlorophenyl)-3–(4-methyl-1-oxo-1H-isochromen-3-yl)-1H-pyrazole-4-carbaldehyde (X10)** Yield:78%; mp: 288°–290 °C; FT-IR (cm^−1^): 3124, 2911 (C-H), 1715 (C = O isocoumarin), 1679 (C = O aldehyde); ^1^H-NMR (DMSO, δ, ppm): 2.41 (s, 3H), 7.65–8.30 (m, 8H), 9.40 (s, 1H), 10.09 (s, 1H) .

### 2.4. General procedure for the synthesis of X(11–20) derivatives

The aldehyde derivatives **X(6–10)** (10 mmol) was dissolved in acetic acid and the barbituric acid/2-thiobarbituric acid (10 mmol) was added over the mixture and stirred overnight at room temperature. Then, the mixture was filtered off and crystallised from ethanol to yield compounds **X(11–20).** The obtained final products were dried under vacuum and fully characterised by FT-IR, ^1^H-NMR, ^13^C-NMR, and melting points.

**5-((3–(1-oxo-1H-isochromen-3-yl)-1-phenyl-1H-pyrazol-4-yl) methylene) pyrimidine-2,4,6(1H,3H,5H)-trione (X11)** Yield: 82%; mp: >300 °C; FT-IR (cm^−1^): 3235, 3081 (NH), 1738, 1722, 1680 (C = O), 1574 (C = N); ^1^H-NMR (DMSO, δ, ppm): 7.44–8.20 (m, 10H), 8.68 (s, 1H), 9.74 (s, 1H), 12.41 (s, 1H, NH), 12.45 (s, 1H, NH).

**5-((1–(4-chlorophenyl)-3–(1-oxo-1H-isochromen-3-yl)-1H-pyrazol-4-yl) methylene) pyrimidine-2,4,6(1H,3H,5H)-trione (X12)** Yield: 81%; mp: >300^o^C; FT-IR (cm^−1^): 3238, 3058 (NH), 1735, 1699, 1666 (C = O), 1571 (C = N); ^1^H-NMR (DMSO, δ, ppm): 7.20–8.20 (m, 9H), 8.62 (s, 1H), 9.69 (s, 1H), 11.29 (s, 1H, NH), 11.36 (s, 1H, NH).

**4–(3-(1-oxo-1H-isochromen-3-yl)-4-((2,4,6-trioxotetrahydropyrimidin-5(2H)-ylidene) methyl)-1H-pyrazol-1-yl) benzonitrile (X13)** Yield: 80%; mp: >300^o^C; FT-IR (cm^−1^): 3192, 3068 (NH), 2231 (-C≡N), 1735, 1712, 1676 (C = O), 1565 (-C = N); ^1^H-NMR (DMSO, δ, ppm): 7.37–8.17 (m, 9H), 8.56 (s, 1H), 9.71 (s, 1H), 11.28 (s, 1H, NH), 11.36 (s, 1H, NH).

**5-((3–(4-methyl-1-oxo-1H-isochromen-3-yl)-1-phenyl-1H-pyrazol-4-yl) methylene) pyrimidine-2,4,6(1H,3H,5H)-trione (X14)** Yield: 81%; mp: >300^o^C; FT-IR (cm^−1^): 3248, 3084 (NH), 1731, 1712, 1686 (C = O), 1568 (C = N); ^1^H-NMR (DMSO, δ d, ppm): 2.46 (s, 3H), 7.59–7.91 (m, 9H), 8.11 (s, 1H), 9.76 (s, 1H), 11.32 (s, 1H, NH), 11.33 (s, 1H, NH). ^13^C-NMR (DMSO, δ, ppm): 13.3, 114.8, 116, 117.2, 120.3, 120.8, 125, 128.8, 129.6, 130, 130.4, 134.8, 136.1, 137.6, 138.8, 142, 142.1, 150.2, 150.5, 161.1, 162.9, 163.6…

**5-((1–(4-chlorophenyl)-3–(4-methyl-1-oxo-1H-isochromen-3-yl)-1H-pyrazol-4-yl) methylene) pyrimidine-2,4,6(1H,3H,5H)-trione (X15)** Yield: 82%; mp: >300^o^C; FT-IR (cm^−1^): 3163, 3042 (NH), 1738, 1709, 1666 (C = O), 1575 (C = N); ^1^H-NMR (DMSO, δ, ppm): 2.46 (s, 3H), 7.63–8.25 (m, 8H), 8.27 (s, 1H), 9.75 (s, 1H), 11.32 (s, 1H, NH), 11.33 (s, 1H, NH).

**5-((3–(1-oxo-1H-isochromen-3-yl)-1-phenyl-1H-pyrazol-4-yl) methylene)-2-thioxodihydropyrimidine-4,6(1H,5H)-dione (X16)** Yield: 82%; mp: >300^o^C; FT-IR (cm^−1^): 3143, 3055 (NH), 1761, 1715 (C = O), 1565 (C = N); ^1^H-NMR (DMSO, δ, ppm): 7.42–8.20 (m, 10H), 8.64 (s, 1H), 9.69 (s, 1H), 11.28 (s, 1H, 1NH), 11. 35 (s, 1H, 1NH). ^13^C-NMR (DMSO, δ, ppm): 107.3, 116.3, 116.5, 120.2, 120.6, 127.4, 128.9, 129.4, 129.9, 130.3, 135.5, 135.9, 136.8, 138.6, 143.9, 147.3, 149, 160.7, 161, 162, 162.7, 178.8.

**5-((1–(4-chlorophenyl)-3–(1-oxo-1H-isochromen-3-yl)-1H-pyrazol-4-yl) methylene)-2-thioxodihydropyrimidine-4,6(1H,5H)-dione (X17)** Yield: 82%; mp: >300^o^C; FT-IR (cm^−1^): 3137, 3075 (NH), 1754, 1712, (C = O), 1558 (C = N); ^1^H-NMR (DMSO, δ, ppm): 7.42–8.18 (m, 9H), 8.65 (s, 1H), 9.72 (s, 1H), 12.39 (s, 1H, 1NH), 12. 45 (s, 1H, 1NH).

**4–(4-((4,6-dioxo-2-thioxotetrahydropyrimidin-5(2H)-ylidene) methyl)-3–(1-oxo-1H-isochromen-3-yl)-1H-pyrazol-1-yl) benzonitrile (X18)** Yield: 80%; mp: >300^o^C; FT-IR (cm^−1^): 3215, 3137 (NH), 2235 (C≡N), 1751, 1715 (C = O), 1574 (C = N); ^1^H-NMR (DMSO, δ, ppm): 2.46 (s, 3H), 7.47–8.26 (m, 9H), 8.28 (s, 1H), 9.79 (s, 1H), 12.43 (s, 2H, 2NH).

**5-((3–(4-methyl-1-oxo-1H-isochromen-3-yl)-1-phenyl-1H-pyrazol-4-yl) methylene)-2-thioxodihydropyrimidine-4,6(1H,5H)-dione (X19)** Yield: 81%; mp: >300^o^C; FT-IR (cm^−1^): 3147, 2902 (NH), 1705, 1666 (C = O), 1568 (C = N); ^1^H-NMR (DMSO, δ, ppm): 2.46 (s, 3H), 7.61–8.26 (m, 9H), 8.28 (s, 1H), 9.78 (s, 1H), 12.43 (s, 2H, 2NH).^13^C-NMR (DMSO, δ, ppm): 13.4, 115, 116.1, 117.4, 120.3, 120.8, 125, 128.9, 129.6, 130, 130.4, 135, 136.1, 137.6, 138.7, 141.9, 143, 150.4, 160.7, 161, 161.9, 172.9, 178.8.

**5-((1–(4-chlorophenyl)-3–(4-methyl-1-oxo-1H-isochromen-3-yl)-1H-pyrazol-4-yl) methylene)-2-thioxodihydropyrimidine-4,6(1H,5H)-dione (X20)** Yield: 82%; mp: >300^o^C; FT-IR (cm^−1^): 3130, 2915 (NH), 1715, 1669 (C = O), 1568 (C = N); ^1^H-NMR (DMSO, δ, ppm): 7.47–8.26 (m, 8H), 8.42 (s, 1H), 9.76 (s, 1H), 11.35 (s, 2H, 2NH).

### 2.5. Ca inhibition assay

An SX.18 MV-R Applied Photophysics (Oxford, UK) stopped-flow instrument has been used to assay the inhibition of various CA isozymes^7^. Phenol Red (at a concentration of 0.2 mM) has been used as an indicator, working at the absorbance maximum of 557 nm, with 10 mM Hepes (pH 7.4) as a buffer, 0.1 M Na_2_SO_4_ or NaClO_4_ (for maintaining constant the ionic strength; these anions are not inhibitory in the used concentration), following the CA-catalyzed CO_2_ hydration reaction for a period of 5–10 s. Saturated CO_2_ solutions in water at 25 °C were used as substrate. Stock solutions of inhibitors were prepared at a concentration of 10 mM (in DMSO-water 1:1, v/v) and dilutions up to 0.01 nM done with the assay buffer mentioned above. At least seven different inhibitor concentrations have been used for measuring the inhibition constant. Inhibitor and enzyme solutions were pre-incubated together for 15 min–6 h at 4 °C prior to assay, in order to allow for the formation of the E-I complex. Triplicate experiments were done for each inhibitor concentration, and the values reported throughout the paper is the mean of such results. The inhibition constants were obtained by nonlinear least-squares methods using the Cheng-Prusoff equation, as reported earlier, and represent the mean from at least three different determinations^1^^,^^8–14^. All CA isozymes used here were recombinant proteins obtained as reported earlier by our group.

## 3. Results and discussion

### 3.1. Chemistry

**Scheme 1.**
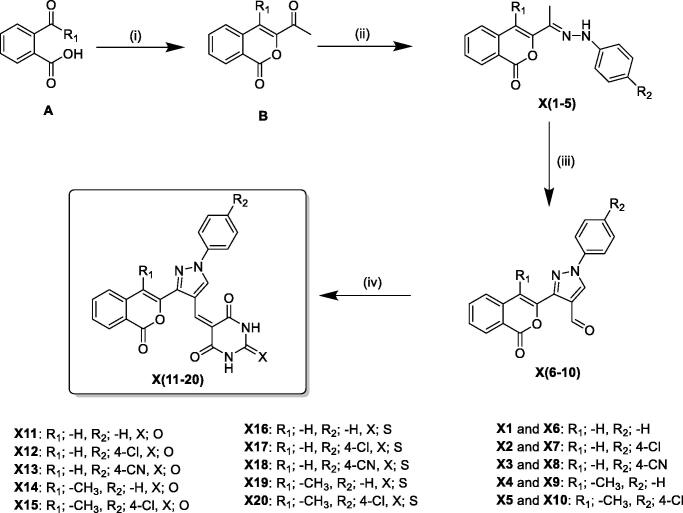
General synthetic route for the synthesis of the isocoumarin-substituted compounds **X(1-20)**. Reagent and conditions: (i) chloroacetone, TEA, 170 °C, (ii) substituted phenylhydrazine hydrochloride, EtOH, sodium acetate, 2 h reflux, (iii) DMF/POCl_3_, 0–5 °C, then 3 h reflux, (iv) barbituric acid/2-thiobarbituric acid, acetic acid.

The structurally diverse isocoumarin derivatives **X(1–20)** were synthesised according to the general synthetic route shown in Scheme 1. 3-Acetylisocoumarin-substituted compounds **B** were synthesised as previously described by some of us^15^. The hydrazone derivatives **X(1–5)** were obtained by reacting **B** with substituted hydrazines^16^. The aldehydes **X(6–10)** were synthesised in high yields by using the Vilsmeier-Haack procedure^17^. These aldehydes were condensed with barbituric acid/2-thiobarbituric acid under acid condition at reflux to produce the final derivatives **X(10–20).** The chemical structures of the novel isocoumarin-substituted derivatives reported here were confirmed by analytical and spectral data (see Materials and methods for details).

### 3.2. Carbonic anhydrase inhibition

Coumarins act as prodrug inhibitors, being hydrolysed by the esterase activity of CAs to the corresponding hydroxy-cinnamic acids which *per se* act as inhibitors, binding at the entrance of the enzyme active site and occluding it^6^. Thus, unlike other inhibitors, such as the anions, the sulphonamides and their isosteres, etc., ^6^, the enzyme and the inhibitor are incubated for at least 6 h in order to allow for the hydrolysis to occur. This was also the protocol that we used for assaying the CA inhibition with isocoumarins, since incubation times of 15 min–3 h led to low but increasing levels of inhibition (data not shown). However, after 6 h incubation, the inhibition levels remained constant and are shown in Table 1.

**Table 1.** Inhibition data of human CA I, II, IX and XII with compounds **X1-20** and the standard sulphonamide inhibitor acetazolamide (**AAZ**) by a stopped-flow CO_2_ hydrase assay^7^. 
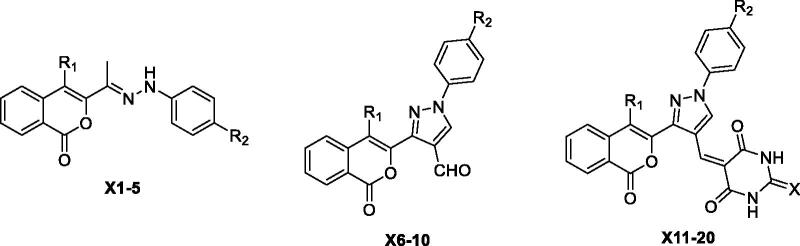

Cmpd.	R_1_	R_2_	X	K_I_ (µM)^a^^,b^			
				hCA I	hCA II	hCA IX	hCA XII
**X1**	-H	-H	–	>100	>100	4.3	1.9
**X2**	-H	-Cl	–	>100	>100	3.5	4.6
**X3**	-H	-CN	–	>100	>100	2.8	1.2
**X4**	-CH_3_	-H	–	>100	>100	3.9	2.3
**X5**	-CH_3_	-Cl	–	>100	>100	2.7	5.8
**X6**	-H	-H	–	>100	>100	7.1	6.5
**X7**	-H	-Cl	–	>100	>100	6.2	11.6
**X8**	-H	-CN	–	>100	>100	8.0	8.3
**X9**	-CH_3_	-H	–	>100	>100	8.9	8.1
**X10**	-CH_3_	-Cl	–	>100	>100	8.1	13.4
**X11**	-H	-H	O	>100	>100	46.2	41.7
**X12**	-H	-Cl	O	>100	>100	>100	>100
**X13**	-H	-CN	O	>100	>100	62.4	36.2
**X14**	-CH_3_	-H	O	>100	>100	54.6	66.5
**X15**	-CH_3_	-Cl	O	>100	>100	>100	>100
**X16**	-H	-H	S	>100	>100	49.7	47.1
**X17**	-H	-Cl	S	>100	>100	>100	>100
**X18**	-H	-CN	S	>100	>100	78.9	53.4
**X19**	-CH_3_	-H	S	>100	>100	72.3	62.8
**X20**	-CH_3_	-Cl	S	>100	>100	>100	>100
**AAZ**	–	–	–	0.250	0.0125	0.026	0.0057

^a^Mean from three different assays, by a stopped flow technique (errors were in the range of ± 5–10% of the reported values).

^b^incubation time 6 h.

Four human (h) CA isoforms, known to be relevant drug targets (hCA I, II, IX and XII)^18^ were included in the work for assessing their inhibition by the isocoumarins reported here (Table 1). It may be observed that as for many coumarins^6^, hCA I and II were not inhibited by isocoumarins up until 100 µM concentrations of inhibitor in the assay system. On the contrary, many isocoumarins (except **X12, X17** and **X20**) showed low micromolar inhibitory power against these isoforms, with K_I_s in the range of 2.7 − 78.9 µM against hCA IX and of 1.2 − 66.5 µM against hCA XII, respectively (Table 1). It can be observed that the less bulky isocoumarins **X1-5** and **X6-10** were the most effective CAIs in the investigated series, with K_I_-s against hCA IX and XII < 15 µM, whereas the compounds incorporating bulkier moieties, such as **X11–20** showed a reduced inhibitory power. This is to be expected, since the active site cavity of these enzymes may not easily accommodate two bulky moieties (phenylpyrazole and pyrimidine-trione/thioxo-pyrimidine-dione) present in some of these compounds.

## 4. Conclusions

We investigated here whether isocoumarins, which are isomeric compounds to comarins known to act as effective CAIs, also act as inhibitors of this enzyme. A series of 3-substituted and 3,4-disubstituted isocoumarins incorporating phenyl-hydrazone, 1-phenyl-pyrazole and pyrazolo-substituted pyrimidine trione/thioxo-pyrimidine dione moieties prepared by an original approach were investigated for their interaction with hCA I, II, IX and XII, known to be important drug targets. hCA I and II were not inhibited by these compounds, whereas hCA IX and XII were inhibited in the low micromolar range by the less bulky derivatives. The inhibition constants ranged between 2.7 − 78.9 µM against hCA IX and of 1.2 − 66.5 µM against hCA XII. As for the coumarins, we hypothesise that the isocoumarins are hydrolysed by the esterase activity of the enzyme with formation of 2-carboxy-phenylacetic aldehydes which act as CA inhibitors. Isocoumarins represent a new class of CAIs.
